# 2196 Pre-treatment sleep disturbance as a risk factor for radiation therapy induced pain in 676 women with breast cancer

**DOI:** 10.1017/cts.2018.177

**Published:** 2018-11-21

**Authors:** Anita R. Peoples, Wilfred R. Pigeon, Dongmei Li, Joseph A. Roscoe, Sheila N. Garland, Michael L. Perlis, Vincent P. Vinciguerra, Thomas Anderson, Lisa S. Evans, James L. Wade, Deborah J. Ossip, Gary R. Morrow, Julie R. Wolf

**Affiliations:** 1 University of Rochester Medical Center; 2 Memorial University; 3 University of Pennsylvania; 4 Northwell Health NCORP, Lake Success, NY, USA; 5 Columbus NCORP, Columbus, OH, USA; 6 Southeast Clinical Oncology Research (SCOR) Consortium NCORP, Winston-Salem, NC, USA; 7 Heartland Cancer Research NCORP, Decatur, IL, USA

## Abstract

OBJECTIVES/SPECIFIC AIMS: The purpose of the present secondary data analysis was to examine the effect of moderate-severe disturbed sleep before the start of radiation therapy (RT) on subsequent RT-induced pain. METHODS/STUDY POPULATION: Analyses were performed on 676 RT-naïve breast cancer patients (mean age 58, 100% female) scheduled to receive RT from a previously completed nationwide, multicenter, phase II randomized controlled trial examining the efficacy of oral curcumin on radiation dermatitis severity. The trial was conducted at 21 community oncology practices throughout the US affiliated with the University of Rochester Cancer Center NCI’s Community Oncology Research Program (URCC NCORP) Research Base. Sleep disturbance was assessed using a single item question from the modified MD Anderson Symptom Inventory (SI) on a 0–10 scale, with higher scores indicating greater sleep disturbance. Total subjective pain as well as the subdomains of pain (sensory, affective, and perceived) were assessed by the short-form McGill Pain Questionnaire. Pain at treatment site (pain-Tx) was also assessed using a single item question from the SI. These assessments were included for pre-RT (baseline) and post-RT. For the present analyses, patients were dichotomized into 2 groups: those who had moderate-severe disturbed sleep at baseline (score≥4 on the SI; n=101) Versus those who had mild or no disturbed sleep (control group; score=0–3 on the SI; n=575). RESULTS/ANTICIPATED RESULTS: Prior to the start of RT, breast cancer patients with moderate-severe disturbed sleep at baseline were younger, less likely to have had lumpectomy or partial mastectomy while more likely to have had total mastectomy and chemotherapy, more likely to be on sleep, anti-anxiety/depression, and prescription pain medications, and more likely to suffer from depression or anxiety disorder than the control group (all *p*’s≤0.02). Spearman rank correlations showed that changes in sleep disturbance from baseline to post-RT were significantly correlated with concurrent changes in total pain (*r*=0.38; *p*<0.001), sensory pain (*r*=0.35; *p*<0.001), affective pain (*r*=0.21; *p*<0.001), perceived pain intensity (*r*=0.37; *p*<0.001), and pain-Tx (*r*=0.35; *p*<0.001). In total, 92% of patients with moderate-severe disturbed sleep at baseline reported post-RT total pain compared with 79% of patients in the control group (*p*=0.006). Generalized linear estimating equations, after controlling for baseline pain and other covariates (baseline fatigue and distress, age, sleep medications, anti-anxiety/depression medications, prescription pain medications, and depression or anxiety disorder), showed that patients with moderate-severe disturbed sleep at baseline had significantly higher mean values of post-RT total pain (by 39%; *p*=0.033), post-RT sensory pain (by 41%; *p*=0.046), and post-RT affective pain (by 55%; *p*=0.035) than the control group. Perceived pain intensity (*p*=0.066) and pain-Tx (*p*=0.086) at post-RT were not significantly different between the 2 groups. DISCUSSION/SIGNIFICANCE OF IMPACT: These findings suggest that moderate-severe disturbed sleep prior to RT is an important predictor for worsening of pain at post-RT in breast cancer patients. There could be several plausible reasons for this. Sleep disturbance, such as sleep loss and sleep continuity disturbance, could result in impaired sleep related recovery and repair of tissue damage associated with cancer and its treatment; thus, resulting in the amplification of pain. Sleep disturbance may also reduce pain tolerance threshold through increased sensitization of the central nervous system. In addition, pain and sleep disturbance may share common neuroimmunological pathways. Sleep disturbance may modulate inflammation, which in turn may contribute to increased pain. Further research is needed to confirm these findings and whether interventions targeting sleep disturbance in early phase could be potential alternate approaches to reduce pain after RT.

